# New gastropod records for the Eastern Mediterranean Sea and one new alien (*Emarginula decorata* Deshayes, 1863) for the Mediterranean Sea from NW Aegean Sea, Greece

**DOI:** 10.1186/2241-5793-21-20

**Published:** 2014-11-27

**Authors:** Thanasis Manousis, Sofia Galinou-Mitsoudi

**Affiliations:** PO Box 48 K, 575 00 Epanomi, Greece; Department of Fisheries & Aquaculture Technology, Alexander Technological Educational Institute of Thessaloniki, 632 00 Nea Moudania, Chalkidiki Greece

**Keywords:** Aegean Sea, Alien species, Biodiversity, Gastropods, Mediterranean Sea, Greece

## Abstract

**Background:**

The NW Aegean Sea has a complex topography, high quality waters, oligotrophic to eutrophic conditions, is connected with estuaries and wetlands, is of high ecological interest, harbours all the types of human activities and yet few researchers work on its marine biodiversity. With this study, the contribution to the knowledge of the Hellenic and Eastern Mediterranean gastropod biodiversity of the studied families is continued, and an expansion of the search in other substrates and deeper waters of the NW Aegean Sea with emphasis on the minor in size species during the period from October 2008 to January 2014.

**Results:**

Thirty seven species belonging to seven families (Cerithiopsidae, Fissurellidae, Phasianellidae, Scissurellidae, Siliquariidae, Skeneidae, and Triphoridae) were identified and their biodiversity was compared with the current checklists of marine gastropod molluscs for the Hellenic Seas based on previous surveys. In this collection of gastropods, one species (*Emarginula decorata* Deshayes, 1863) is a new alien for the Mediterranean Sea, 14 species are new for the Eastern Mediterranean Sea and 16 species are new for the Hellenic fauna (with the one above mentioned alien species included). The main identification characteristics and ecological information such as habitat, distribution, alien expansion paths to the NW Aegean Sea and origin of the species are given and discussed.

**Conclusions:**

The Hellenic gastropod biodiversity of the studied families was enriched with 37 new records for the N Aegean Sea, out of which 16 are new for Greece, 14 are new for the Eastern Mediterranean Sea while one (*Emarginula decorata*) is a new alien for the Mediterranean Sea.

## Background

**Figure 1:**
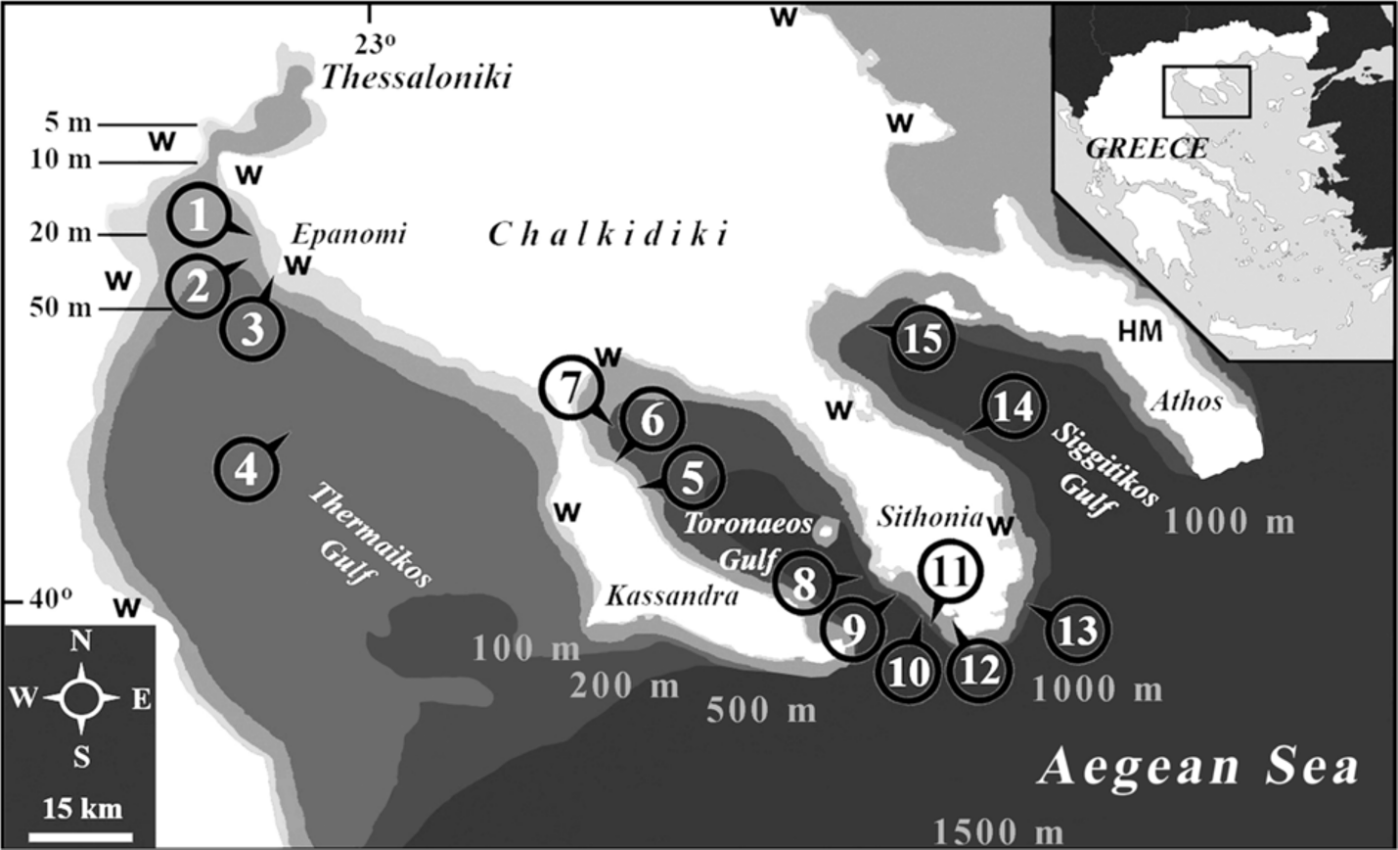
**Map of studied area.** Stations in Thermaikos Gulf: 1. Palioura; 2. Paralia of Epanomi; 3. Cape of Epanomi; 4. Central Thermaikos Gulf. Stations in Chalkidiki: 5. Afitos; 6. Nea Fokaia; 7. NW Toronaeos Gulf; 8. Spalathonisia; 9. Tristinika; 10. Marathia; 11. Toroni; 12. Porto Koufo; 13. Ampelos; 14. Central Siggitikos Gulf; 15. Pyrgadikia. The protected by NATURA 2000 wetland areas (w) as well as the Holy Mount of monaster life and unique flora in Athos peninsula (HM), are also indicated.

The N Aegean Sea comprises a marginal sea that connects the Eastern Mediterranean basin with the Black Sea through the Dardanelles Strait. Its North West (NW) area exhibits a complex topography that includes estuarine areas, long gulfs and peninsulas of both shallow and deep, and high quality waters (51 or 13% of the 393 blue flags of Greece, 2^nd^ world rank for 2013 by Blue Flag [1] of depths up to 1500 m in the western part of the N Aegean Trough [2] (Figure 1)). The area receives significant loads of riverine nutrients and low-salinity productive waters from the Black Sea. In the surface of the open sea (around the peninsulas of Chalkidiki as well as in the Toronaeos Gulf), the concentration of chl-a is low (0.2 μg l^-1^) rising to eutrophic levels by the estuaries while its values are much higher (0.4 μg l^-1^) close to the bottom [3].

Thermaikos Gulf (with the second big for Greece City and Port of Thessaloniki) is affected by the riverine loads and the human activities (agriculture, mussel culture, fishery and industry at the N and W coasts; tourism and navigation at the E and SW of 1250000 people) [4].

In between the three Peninsulas of Chalkidiki, the Toronaeos and Siggitikos Gulfs are deep marine basins of high quality waters with coasts of variable type from calm and sandy to exposed rocky cliffs; 70000 people live permanently in that area employed in jobs related to tourism and agriculture while in the summer that number increases sharply with the tourists to an estimated 1200000 [4].

As a whole, the ecological importance of the NW Aegean coastal zones is based upon the high quality of the sea water, the small or extended wetlands (all protected by NATURA 2000 and Ramsar Convention) and the Mount Athos Peninsula (Figure 1) with its virgin forests and the incredible floristic biodiversity all aided by the monastic life of its inhabitants. In addition, NW Aegean comprises one of the main oil tanker and cargo ship traffic routes of the E Mediterranean Sea [5] - known vectors for the transfer of alien organisms.

After a long and slow cooling period from the late 1960s to the early 1990s, Aegean Sea started to warm rapidly. The warming rate over 1992–2008 was several times higher than the estimated global mean warming rate over the same period [6]. During the early 1990s, a significant change in the E Mediterranean thermohaline circulation was observed, that of the shift of dense waters from the Adriatic Sea to the Aegean Sea, a phenomenon now known as the “Eastern Mediterranean Transient” [3]. These environmental changes were not followed by extensive faunistic studies and thus their impact on the biodiversity of the area was not assessed. Only few and sporadic investigations are referred to the gastropod fauna of the Hellenic Seas - mainly as a part of faunistic research (e.g. [7–12]) - and fewer are referred to the NW Aegean Sea with most recent those of Manousis *et al*. [13] and among the previews publications by Sakellariou [7], Tenekidis [8], Koutsoubas *et al*. [9], Antoniadou *et al*. [10] and Koroneos [14]. At the same time, it is known that progress in benthos research, human activities and environmental conditions change significantly the recorded marine biodiversity while detailed and persisting surveys usually increase the number of the recorded species, mainly of those of small size [15–18].

The aim of this study was: a) to continue contributing to the knowledge of the Hellenic gastropod biodiversity with an updated list for the gastropod species of the studied families from the NW Aegean Sea, and b) to expand the research effort in other substrates and deeper waters.

## Results

### The records

**Figure 2:**
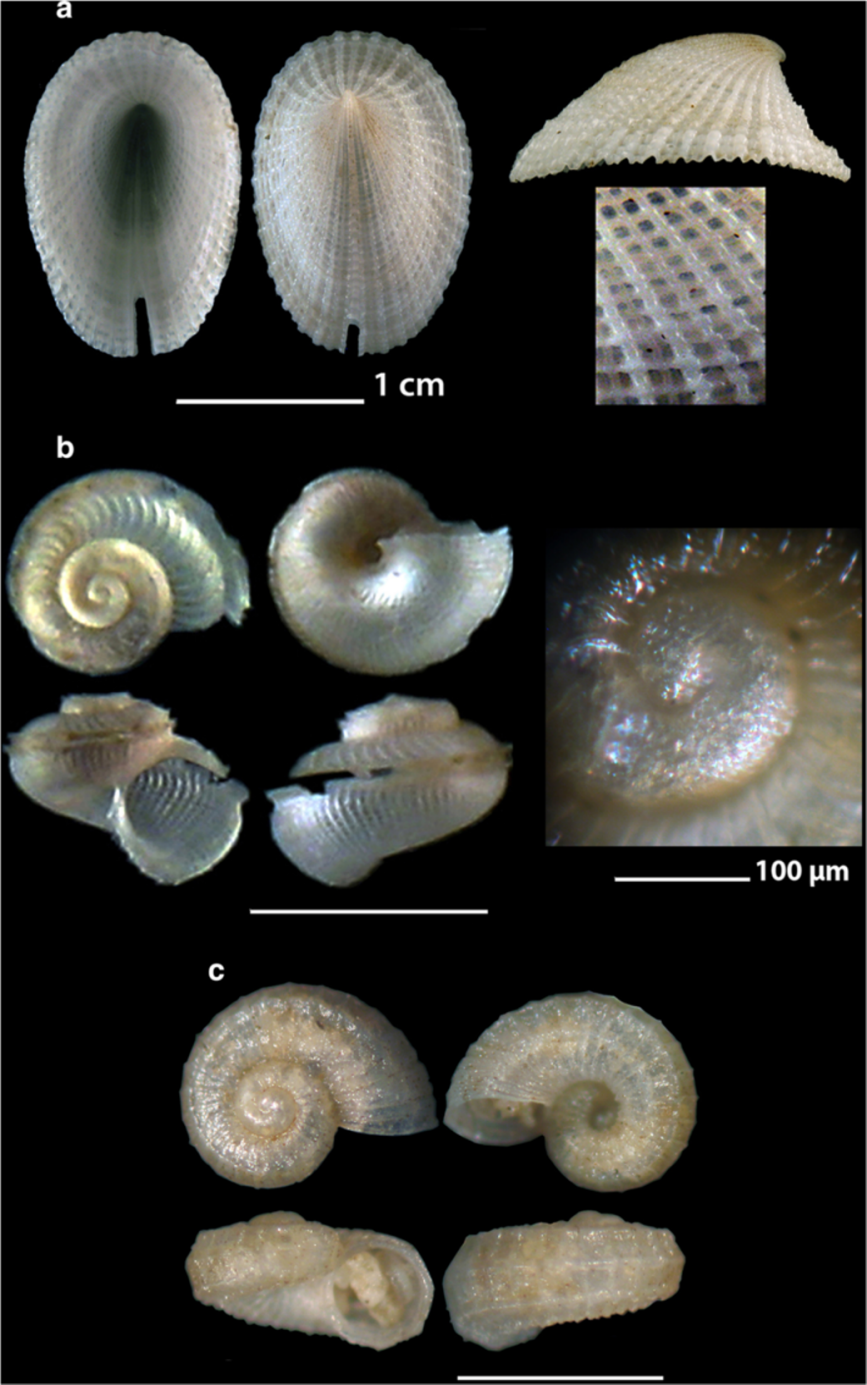
**The new recorded species of the families Fissurellidae: a.** ***Emarginula decorata*** **, Scissurellidae. b.** ***Anatoma micalii*** **and Skeneidae:** **c.** ***Skeneoides exilissima.*** Bar = 1 mm.

**Figure 3:**
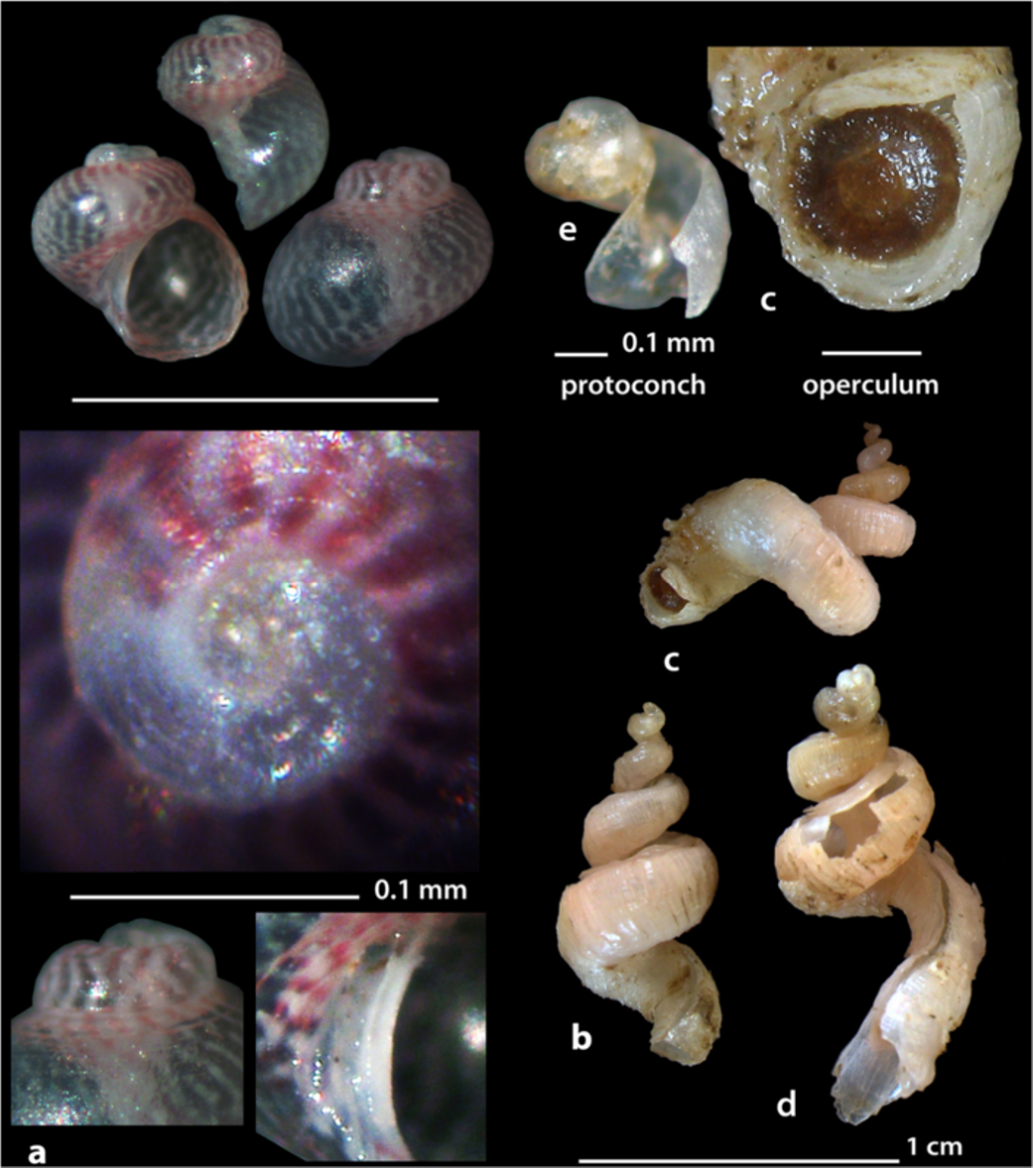
**The new recorded species of the families Phasianellidae: a.** ***Tricolia deschampsi*** **and Siliquariidae:** **b, c, d** **and e.** ***Petalopoma elisabettae.*** Bar = 1 mm (unless otherwise indicated).

**Figure 4:**
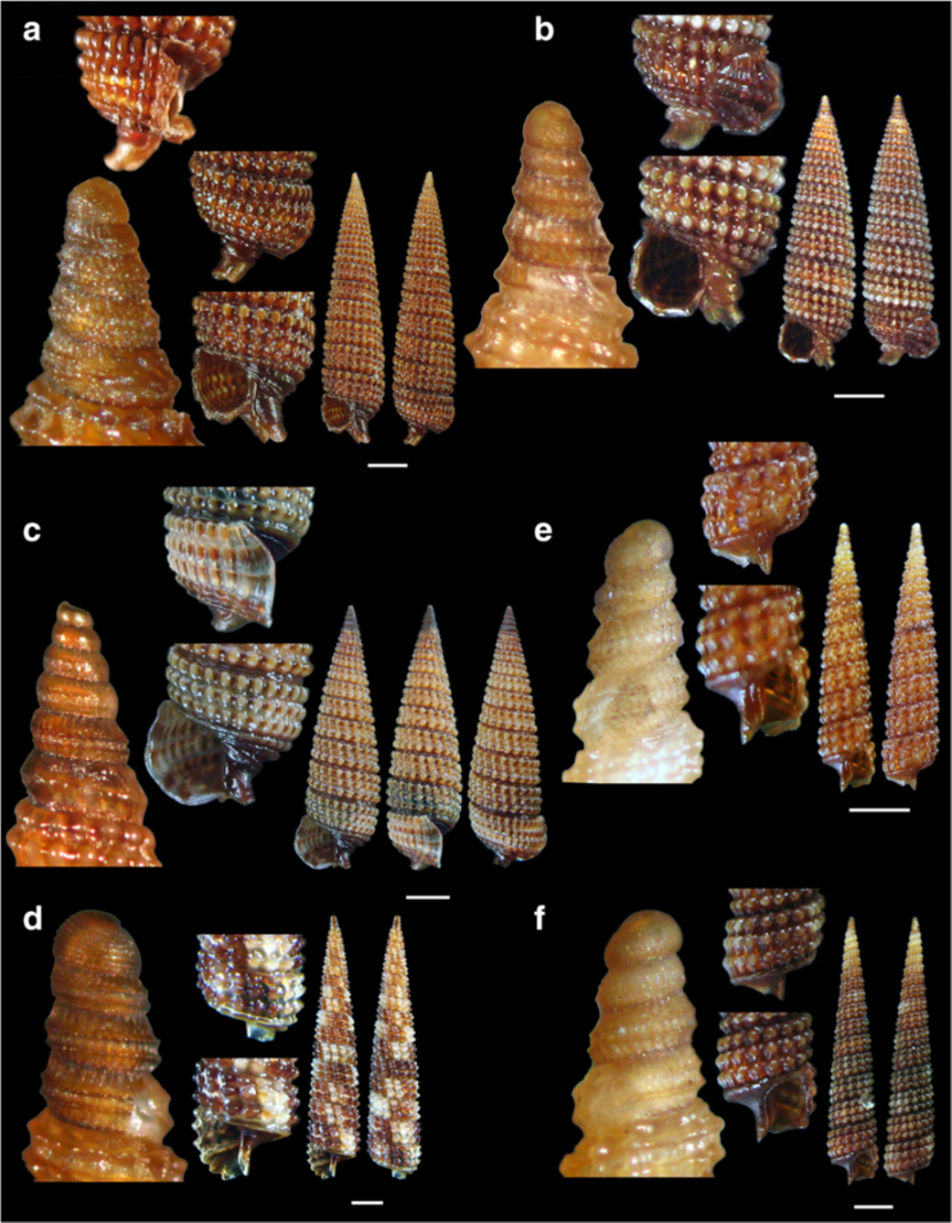
**The new recorded species of the families Triphoridae: a.** ***Monophorus thiriotae*** **, b.** ***Obesula marinostri*** **, c.** ***Similiphora triclotae*** **, d.** ***Strobiligera flammulata*** **, and Cerithiopsidae** **: e** **and f.** ***Cerithiopsis atalaya.*** Bar = 1 mm.

**Figure 5:**
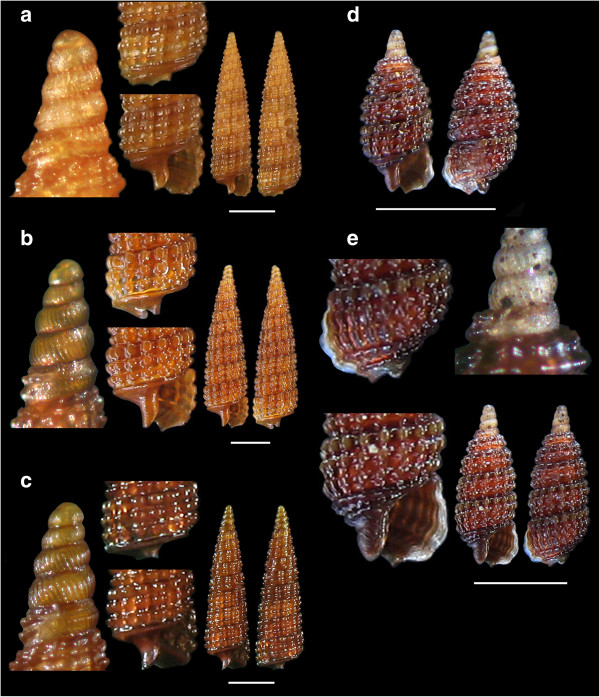
**The new recorded species of the family Cerithiopsidae: a.** ***Cerithiopsis diadema*** **, b** **and c.** ***Cerithiopsis fayalensis*** **, d** **and e.** ***Cerithiopsis micalii.***

**Figure 6:**
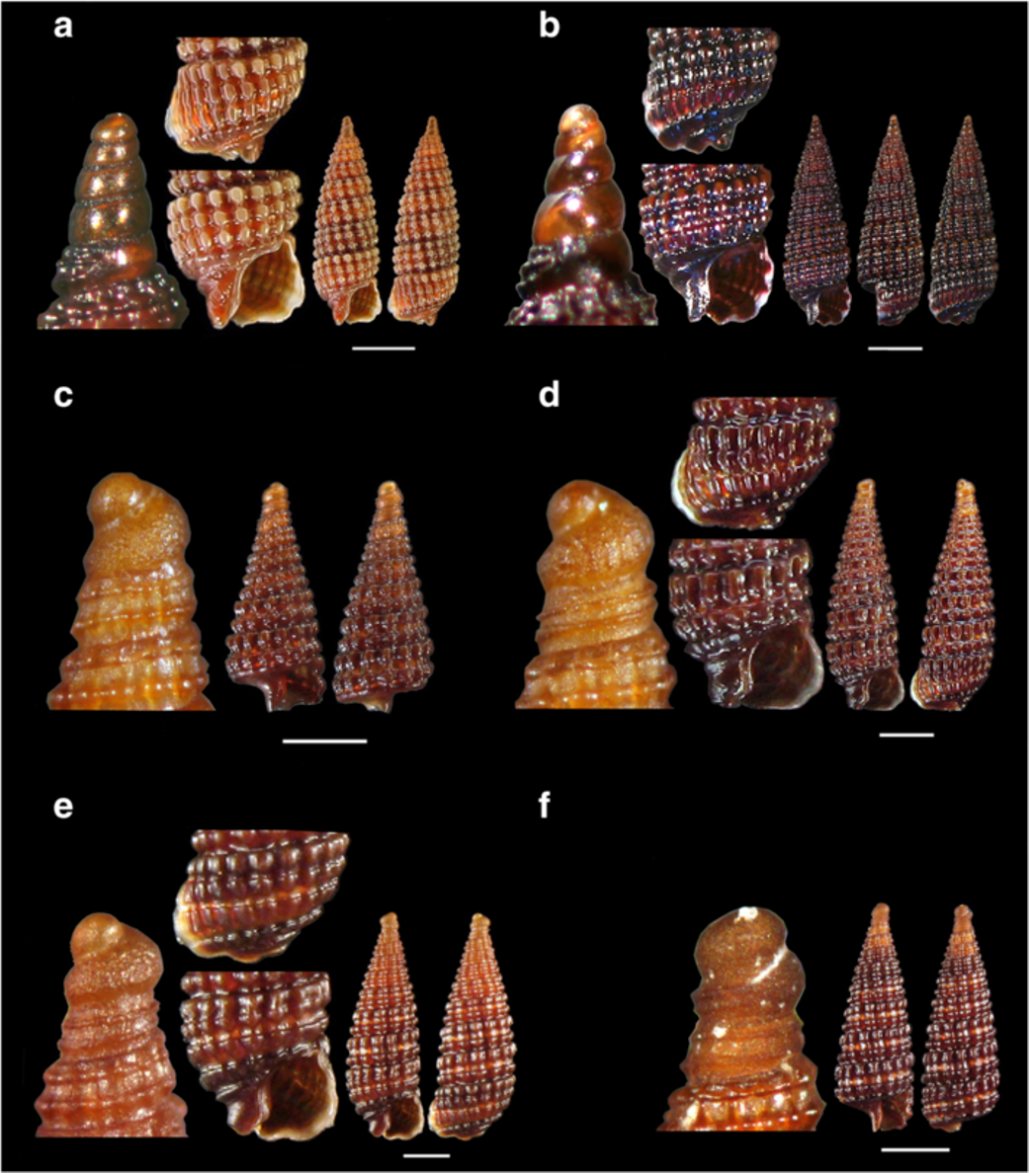
**The new recorded species of the family Cerithiopsidae: a.** ***Cerithiopsis nana***, **b.** ***Cerithiopsis cf. oculisfictis*** **, c, d, e** **and f.** ***Dizoniopsis concatenata.*** Bar = 1 mm.

**Figure 7:**
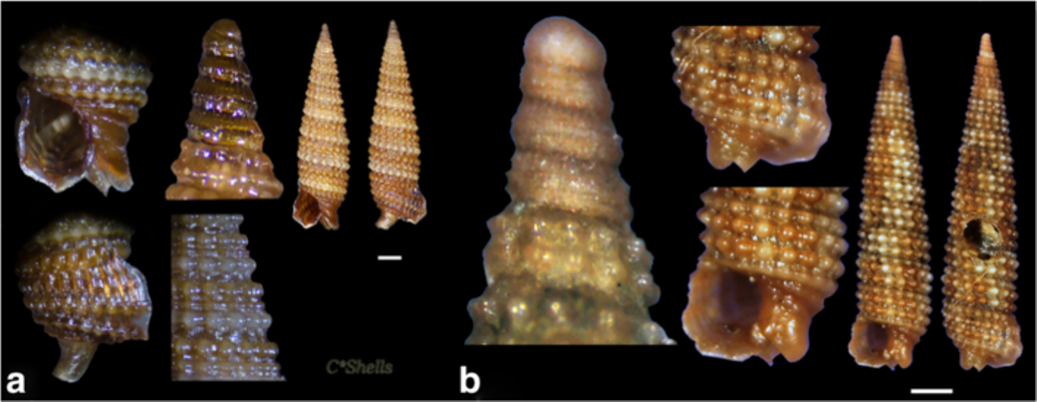
**Additional samples collected and photographed by C. Kontadakis: a.** ***Obesula marisnostri*** **and G. Mpazios** **: b.** ***Strobiligera flammulata.*** Scale bars = 1 mm.

As a result of this investigation, approximately 150 specimens were collected and 37 species were identified. They are listed within families in Table 1 and presented in Figures 2, 3, 4, 5, 6 and 7. Among the identified species, 14 are recognized as new for the gastropods fauna of E Mediterranean Sea and 16 are referred for the first time to the Hellenic fauna, one of which is alien. The alien is *Emarginula decorata* Deshayes, 1863 was referred from the SE Africa, Reunion Islands and Red Sea and is recorded for the first time from the Mediterranean Sea. All species with data on their habitat, mode of life and origin are presented in Table 1.

**Table 1:** **Gastropods records, habitat and distribution details (in the study area)**

FAMILY (the change of species number/genus, % additions for Greece based on Koukouras [19] and Manousis ***et al*** . [17] )	Species	New record R: Greece EM: E Medit. A: Alien in Medit.	The collection Stations in the Gulfs			Zone/Depth (m)	Habitat	Mode of life [20]	Found	Origin
			Thermaikos (1–4 stations)	Toronaeos (5–12 stations)	Siggitikos: (13–15 stations)					
FISSURELLIDAE	*Emarginula adriatica* Costa, 1829		1	9, 10	14, 15	10 - 120	*Posidonia,* & mixed bottom	Feeds on sponges	Alive & shells	Mediterranean Sea
(6 species from 5, 20% for the genus)	*Emarginula decorata* Deshayes, 1863	R, EM, A			14	120	Mixed bottom	Feeds on sponges	Alive	Red Sea & SE Africa
	*Emarginula huzardii* (Payraudeau, 1826)		1, 3	5, 7, 9, 12	14	60	*Zostera,* biogenic & mixed bottom	Feeds on sponges	Alive & shells	Mediterranean Sea
	*Emarginula octaviana* Coen, 1939				15	10 - 45	Biogenic & mixed bottom	Feeds on sponges	Alive	Mediterranean Sea
	*Emarginula rosea* Bell, 1824		1	8, 9, 12	14	10 - 60	Biogenic & mixed bottom	Feeds on sponges	Shells	Mediterranean Sea
	*Emarginula sicula* Gray, 1825		3			9	Mixed bottom	Feeds on sponges	Alive & shell	Mediterranean Sea
SCISSURELLIDAE (new genus for Greece)	*Anatoma micalii* Geiger, 2012	R, EM			14	120	Mixed bottom	Euryphagous	Shells	W Mediterranean Sea & Sea of Marmara
No change	*Scissurella costata* d' Orbigny, 1824		1, 2	7, 8, 11	14	10 - 70	*Posidonia* & biogenic bottom	Feeds on live plant matter	Alive & shells	Mediterranean Sea
SKENEIDAE (new genus for Greece)	*Skeneoides exilissima* (Philippi, 1844)	R, EM	1			18	Mixed bottom	Feeds on hydroids	Shell	W Mediterranean Sea
PHASIANELLIDAE	*Tricolia pullus* (Linnaeus, 1758)		1, 3	5	15, 14	0 - 120	*Zostera,* biogenic & mixed bottom	Feeds on marine algae and grasses	Alive & shells	Mediterranean Sea
(4 species from 3, 33% for the genus)	*Tricolia deschampsi* Gofas, 1993	R, EM			14	120	Mixed bottom	Feeds on marine algae and grasses	Shell	Mediterranean Sea
	*Tricolia speciosa* (Von Muehlfeldt, 1824)		3		15	0 - 30	Mixed bottom	Feeds on marine algae and grasses	Alive & shells	Mediterranean Sea
	*Tricolia tenuis* (Michaud, 1829)		1	5	14, 15	5 - 60	*Zostera,* biogenic & mixed bottom	Feeds on marine algae and grasses	Alive & shells	Mediterranean Sea
SILIQUARIIDAE (new genus for Greece)	*Petalopoma elisabettae* Schiaparelli, 2002	R, EM	1, 4	10	15	10 - 70	Biogenic & mixed bottom	Feeds on sponges	Alive & shells	Central Mediterranean Sea
No change	*Tenagodus obtusus* (Schumacher, 1817)		1			10	Mixed bottom	Feeds on sponges	Shells	Mediterranean Sea
TRIPHORIDAE	*Marshallora adversa* (Montagu, 1903)		3, 4			60 - 70	Mixed bottom	Feeds on sponges	Shells	Mediterranean Sea
No change										
(3 species from 2, 50% for the genus)	*Monophorus erythrosoma* (Bouchet & Guillemot, 1978)		1		14	5 - 120	Mixed bottom	Feeds on sponges	Alive & shells	Mediterranean Sea
	*Monophorus perversus* (Linnaeus, 1758)		3			0	*Zostera*	Feeds on sponges	Shell	Mediterranean Sea
	*Monophorus thiriotae* Bouchet, 1985	R	4		15	30 - 70	Mixed bottom	Feeds on sponges	Alive & shells	Mediterranean Sea
(new genus for Greece)	*Obesula marinostri* Bouchet, 1985	R, EM	1			10	Mixed bottom	Feeds on sponges	Alive	W Mediterranean Sea
(2 species from 1, 100% for the genus)	*Similiphora similior* (Bouchet & Guillemot, 1978)		1, 2, 3, 4	6		0 - 70	*Zostera,* biogenic & mixed bottom	Feeds on sponges	Alive & shells	Mediterranean Sea
	*Similiphora triclotae* (Bouchet, 1996)	R, EM	2		14	40 - 60	Mixed bottom	Feeds on sponges	Alive & shells	W Mediterranean Sea
(new genus for Greece)	*Strobiligera flammulata* Bouchet & Waren, 1993	R, EM			14, 15	30 - 120	Mixed bottom	Feeds on sponges	Alive & shell	SW Mediterranean Sea
CERITHIOPSIDAE	*Cerithiopsis atalaya* Watson, 1885	R, EM	1, 4		15	10 - 70	Mixed bottom	Feeds on sponges	Shells	W Mediterranean Sea
(11 species from 3, 267% for the genus)	*Cerithiopsis barleei Jeffreys, 1867*		1		15	5 - 30	Mixed bottom	Feeds on sponges	Alive & shells	Mediterranean Sea
	*Cerithiopsis diadema* Monterosato, 1874	R, EM			14	60	Mixed bottom	Feeds on sponges	Shell	W & Central Mediterranean Sea
	*Cerithiopsis fayalensis* Watson, 1886	R, EM	1		14	20 - 120	Mixed bottom	Feeds on sponges	Alive & shells	W Mediterranean Sea
	*Cerirhiopsis horrida* (Monterosato, 1874)		4			70	Mixed bottom	Feeds on sponges	Shell	Mediterranean Sea
	*Cerithiopsis micallii* (Cecalupo & Villari, 1997)	R, EM	4			70	Mixed bottom	Feeds on sponges	Alive & shell	W & Central Mediterranean Sea
	*Cerithiopsis minima* (Brusina, 1865)		3		14, 15	0 - 30	Mixed bottom	Feeds on sponges	Alive & Shells	Mediterranean Sea
	*Cerithiopsis nana* Jeffreys, 1867	R	1		15	5 - 50	Mixed bottom	Feeds on sponges	Alive & shell	Mediterranean Sea
	*Cerithiopsis cf. oculisfictis* Prkic & Mariottini, 2010	R, EM	4			100	Mixed bottom	Feeds on sponges	Alive	Mediterranean Sea
	*Cerithiopsis scalaris* (Locard, 1892)		1			10	Mixed bottom	Feeds on sponges	Shell	Mediterranean Sea
	*Cerithiopsis tubercularis* (Montagu, 1803)		1, 2, 4			5 - 70	Mixed bottom	Feeds on sponges	Alive & shells	Mediterranean Sea
(2 species from 1, 100% for the genus)	*Dizoniopsis concatenata* (Conti, 1864)	R, EM	1			20 - 30	Mixed bottom	Feeds on sponges	Alive & shells	Central Mediterranean Sea
	*Dizoniopsis coppolae* (Aradas, 1879)		1, 4			20 - 70	Mixed bottom	Feeds on sponges	Shells	Mediterranean Sea
No change	*Metaxia metaxa* (delle Chiaje, 1828)		1		13, 14, 15	10 - 70	Biogenic & mixed bottom	Feeds on sponges	Alive & shells	Mediterranean Sea

### Documented first records for the Hellenic waters

The specimens’ descriptions per family with basic eco-geographical information for the new records are given below:

#### Fissurellidae

*Emarginula decorata* Deshayes, 1863 (synonym of *E. spinosa*) (Figure 2a).

**Collection station** One live individual (16.55 mm long, 11.35 mm wide and 7.80 mm high) was found on a sponge collected from mixed bottom at 120 m depth of station 14.

**Description** The strong and oval shell has a moderately high profile, a very small slightly coiled apex located at the posterior 1/5 and a slightly curved base that makes it rest on its anterior and posterior margins. The sculpture consists of 55–57 alternating strong and weaker radial ribs more or less of equal strength by the posterior margin and interspaces equally wide as the ribs. High co-marginal cords form tubers when passing over the ribs and produce a prominent lattice. Wide and rectangular slit reaching 1/8 the distance between the anterior margin and the apex. Color; yellowish white.

**Similar species** At first impression, *E. decorata* looks similar to some native Mediteranean species. Nevertheless, although it is of a similar oval shape as *E. christiaensi* Piani, 1985 it has a lower profile and a more prominent sculpture; it has a less coiled apex and more prominent sculpture than *E. sicula* JE Gray, 1825 with the ratio of the length of the fissure to the distance to the shell’s apex to be 1/9 in *E. spinosa*[21], while it is <1/5 in *E. sicula* according to Gofas *et al*. [22]; it is much lower and more oval than *E. fissura* (Linnaeus, 1758) and it is of more oval shape and higher than *E. octaviana* Coen, 1939.

**Habitat and distribution** It was found live foraging on a sponge. Its known distribution is from SE Africa [23], Reunion, Arabian Gulf and Red Sea [21].

**Status** *Emarginula decorata* Deshayes, 1863 is recently referred as a synonym of *E. spinosa* Deshayes, 1863 [21, 24, 25] while the World Register of Marine Species [23] accepts *Emarginula spinosa* Deshayes, 1863 as a separate species from *E. decorata* Deshayes GP, 1863.

#### Scissurellidae

*Anatoma micalii* Geiger, 2012 (Figure 2b).

**Collection station** Four shells (0.75–0.85 mm long and 0.85–1.00 mm wide) were found in detritus material trapped in small scale fishing nets at 120 m depth from mixed bottom of station 14.

**Description** The very delicate, biconic, pearly white and rather depressed in its outline shell is formed by a globose spiral of two finely reticulate convex whorls that are flattened above and rapidly enlarging. The protoconch is approximately 3/4 of a whorl succeeded by the teleoconch I that bears 17–18 axial ribs in 3/4 of a whorl and exhibits an elevation of the spire by the upper outer lip immediately before the onset of the selenizone of the teleoconch. The teleoconch II sculpture consists of numerous fine, curved and longitudinal axial ribs, interrupted by a long furrow with raised margins (selenizone) and intersected by dense minute spiral striae in the interstices. Six of those spiral striae on the upper part (roof) of the shell over the selenizone stand out as they are more robust than the rest. The furrow lies at nearly one-third of the whorl’s height from the suture and is deep, striated across and with sharp and prominent edges forming a peripheral carina. The aperture is oblique and the peristome continuous with a thin outer lip. The inner lip is folded back on the columella without covering the umbilicus. The umbilicus is deep, funnel-shaped, exposing only the body whorl and bearing a very prominent funiculus which starts from behind the edge of the inner lip.

**Similar species** Based on descriptions and detailed figures of Geiger [26] and the *Anatoma micalii* specimens of this study, *A. micalii* is similar, though smaller by half to *A. aspera* (Philippi, 1844) (an inhabitant of the Greek Seas) but differs from the late in that a) it has proportionally more wide the two carinas of the selenizone, b) it has a more rapidly enlarging and, hence, more compressed spire, c) it has six prominent spiral striae on its upper part of the dome, d) its inner apertural lip does not cover the umbilicus, e) it has a more prominent funiculus by the umbilicus and f) it exhibits an elevation of the spire by the upper outer lip immediately before the onset of the selenizone.

**Habitat and distribution** It lives on muddy-detrital bottoms of the circalittoral zone. The species has a rather wide distribution as it has been referred from Angola, Morocco (Strait of Gibraltar), the south coast of France, the west and south coasts of Italy and from the Bosporus [26].

**Status** It is a recently described new species by Geiger [26] who has re-identified erroneous references of *Anatoma* species from the Mediterranean Sea.

#### Skeneidae

*Skeneoides exilissima* (Philippi, 1844) (Figure 2c).

**Collection station** One shell (0.60 mm long, 1.20 mm wide) was found in detritus material trapped in small scale fishing nets at 18 m depth from mixed bottom of station 1.

**Description** The pearly and uniformly white shell is almost planispiral and with a wide and deep umbilicus. Sculpture of 3 spiral cords per whorl visible on the spire, 4 evenly spaced spiral cords on the body whorl plus one on the umbilical wall; axial sculpture of fine, sharp, widely spaced and at equal interspaces axial ribs intersecting with the spiral cords and forming squarish pits. The aperture is circular, continuous and, due to the prominent spiral cords, with a honey comb cell-like periphery. The outer lip is simple.

**Similar species** Resembles *Parviturbo fenestratus* (Chaster, 1896) from which it differs in its much lower spire and therefore the more depressed profile and the much wider umbilical region [27].

**Habitat and distribution** West Mediterranean Sea [21] up to the northern part of the Central Mediterranean Sea [27].

**Status** Less frequent [21] to common [27].

#### Phasianellidae

*Tricolia deschampsi* Gofas, 1993 (Figure 3a).

**Collection station** One shell (0.65 mm long, 0.65 mm wide) was found in detritus material from mixed bottom of station 15.

**Description** The shell has over two whorls and a low spire with an ample body whorl. Its protoconch consists of nearly one whorl, is nearly 90 μm wide and with a strong sculpture decoration. The first quarter of a whorl of the teleoconch bears a spiral microsculpture of 6–7 spiral cords, narrower than the interspaces, gradually replaced on later whorls by spiral rows of small punctures. Umbilical chink narrow, bordered by a sharp and white keel. The aperture is broad and rounded. The protoconch is opaque white. The first quarter of a whorl of the teleoconch is also white and the rest with pink, narrow and sharp lines arranged parallel to the growth lines and opaque white anastomosing lines on a translucent background. The periumbilical area exhibits a wavy contour of alternating pink and white patches.

**Similar species** With slightly smaller dimensions, this species is morphologically similar to the congeneric *Tricolia tingitana* with which it shares the same shape of the umbilicus but has a different color pattern [28]. *T. deschampsi* is also similar to *T. entomocheila* but the late is different in the color pattern and its direction, the shell’s outline and its protoconch lacks the sculptural decoration present in our specimen. Moreover the specimen is dissimilar to *T. pullus* because of the color pattern, the shell’s outline and because *T. pullus* protoconch lacks a sculptural decoration [28].

**Habitat and distribution** Lives in the infralitoral zone (<40 m) between photophilic algae [28, 29]. Species originally known from the area of the Strait of Gibraltar but subsequently collected in several other locations in the Mediterranean [27–29].

**Status** Uncommon [27].

#### Siliquariidae

*Petalopoma elisabettae* Schiaparelli, 2002 (Figures 3b, c,d and e).

**Collection station** Two juvenile shells (2.00 mm and 5.00 mm long, 0.70 mm and 1.60 mm wide, respectively) were found in detritus material trapped in small scale fishing nets at 10 m depth from mixed bottom of station 1. One live individual (9.60 mm long, 0.30 mm wide) still with its operculum and three shells (7.10 mm long, 0.20 mm wide; 11.15 mm long, 2.90 mm wide; 14.00 mm long, 2.90 mm wide) were trawled trapped in a piece of sponge from 70 m at station 4. One shell (4.00 mm long, 1.30 mm wide) was found in detritus material trapped in small scale fishing nets at 30 m depth from mixed bottom of station 15.

**Description** The shell-shaped tubes (characteristic feature of the family) are small and slender, some with an intact lecithotrophic white protoconch and with a side slit of irregular width. The shell bears very irregular and alternating narrow and wider zones. The general outlook of the shell resembles the tip of a spear. The color is light cream-yellow to light cream-pink still retaining in places a brown periostracum.

**Similar species** Very similar in its general outlook to *Tenagodus obtusus* (Schumacher, 1817) from which it differs in its smaller size, its more slender and wiry tube (*T. obtusus* is more depressed and hunched over with the first few laps forming a regular spiral and then taking a variable form), its lecithotrophic protoconch (in contrast to the planktotrophic one of *T. obtusus*), its more irregular in width side slot (in *T. obtusus* it is of a constant width), the alternating narrower and wider zones in the total length of the tube and in its cream color (in contrast to the brown of *T. obtusus*). *Petalopoma elizabetae* seems to be strongly associated with sponges upon which it feeds while *T. obtusus* lives attached to rocks [30].

**Habitat and distribution** It has been found live in sponges from 20 to 40 m of depth in the Central Mediterranean Sea [30] and the W Mediterranean Sea [22].

**Status** Rare [27].

#### Triphoridae

*Monophorus thiriotae* Bouchet, 1985 (Figure 4a).

**Collection station** Two live individuals (7.15 mm and 6.85 mm in length, 1.65 mm and 1.55 mm wide, respectively) and one immature shell (8.95 mm in length, 1.95 mm wide) were collected from detritus material trapped in small scale fishing nets at 70 m depth from mixed bottom of station 4. One shell (8.80 mm in length, 1.90 mm wide) was collected from detritus material trapped in small scale fishing nets at 30 m depth from mixed bottom of station 15.

**Description** The rather slender sinistral shell has a high and regularly conical spire. Its protoconch is pointed, with 4 3/4 moderately convex whorls. The embryonic part is decorated with little tubercules that give the appearance of a reticulated sculpture all over its surface, while the larval part bears two strong carinas that are narrower than their interspace, and axial slightly sigmoid riblets. The teleoconch has 12 almost flat whorls. The sculpture consists of smooth and rounded pearl-like tubercules that are much wider than the interspaces and are aligned axially. In the first whorl of the teleoconch there are two cords, with one third sandwiched in between them much later and increasing in thickness, without becoming equally thick with the others until the last whorl. The last whorl narrows sharply at its base and bears one more granular cord (4th) in prolongation of the suture and two others (the 5th is rough and the 6th slightly wavy) between that and the siphonal canal. In the final part of the last whorl and in the vicinity of the outer lip, the four uppermost cords are not divided into additional cords but simply become flatter. The aperture has an expanded simple outer lip with a fragile edge. The siphonal canal is short, almost closed forming a conical projection and the upper part of the aperture has a deep notch near the suture. The chestnut brown color is lighter on the tubercules while the interspaces are darker.

**Similar species** *Monophorus thiriotae* is quite similar to other members of the family. It differs from the congeneric *M. erythrosoma* (Bouchet & Guillemot, 1978) by not being monochromatic and by the spiral cords not dividing before the end of the spire [31], from *Cheirodonta palescens* (Jeffreys, 1867) by having cords not subdivided on its outer lip and from *Marshallora adversa* (Montagu, 1803) by being plumper and a bit bigger. It also differs from *Cheirodonta*, *Marshallora*, and *Similiphora* in that its additional cords of the last whorl are granular [22].

**Habitat and distribution** In infralittoral rocky bottoms on sponges. Atlantic coast of Europe from the Basque Country to Morocco, the Canary Islands, the Azores and the W Mediterranean Sea [22].

**Status** Uncommon in the W Mediterranean [27].

*Obesula marisnostri* Bouchet, 1985 (Figure 4b).

**Collection station** One live individual (5.50 mm in length, 1.50 mm wide) was collected from detritus material trapped in small scale fishing nets at 20 m depth from mixed bottom of station 1. One more (second) record by the collector Mr. Costantinos Kontadakis from S Greece is discussed in the relevant section.

**Description** The sinistral shell has a high and regularly conical spire. The protoconch is formed by four moderately convex whorls, the first of which is covered with tiny granules and followed by numerous axial ribs interrupted by a single keel up to the beginning of the teleoconch. The late is formed by 10 almost flat whorls separated by a deep suture which is highlighted in relation to the interspaces between the spiral cords. The sculpture consists of spiral cords wider than the interspaces and formed by smooth and rounded pearl-like tubercules which are aligned axially. In the first whorl of the teleoconch there are only two cords, with a third one sandwiched in between them from the fourth whorl and growing rapidly to become of even thickness with the two others. The last whorl is tapered at its base, with an additional granular cord in prolongation of the suture and two more cords between that fourth cord and the siphonal canal. Aperture with a simple, somewhat expanded outer lip and a deep notch near the suture. A thick siphonal canal opened only by a narrow slit and forming a conical projection. The shell is pale chestnut in color, with slightly darker the protoconch, the adapical part of the teleoconch and the tip of the siphonal canal and with slightly lighter the pearls of the third cord.

**Similar species** This species is very easily recognized by the single keel on the protoconch and the two additional granulated cords in the last whorl, in contrast to the smooth or slightly wrinkled ones of the other species [22].

**Habitat and distribution** In funds between 30 m and 200 m of the circalittoral gravel floor of the Atlantic and the Western Mediterranean from the Alboran Sea to Italy, France and Algeria [27].

**Status** Uncommon in the W Mediterranean Sea [22, 27].

*Similiphora triclotae* (Bouchet, 1996) (Figure 4c).

**Collection station** Two live individuals (6.80 mm and 5.00 mm in length, 2.10 mm and 1.70 mm wide, respectively) and two shells (8.00 mm and 5.00 mm in length, 2.55 mm and 2.10 mm wide, respectively) were collected from detritus material trapped in small scale fishing nets at 40 m depth from mixed bottom of station 2.

**Description** The sinistral shell has a high and regularly conical spire. The protoconch is pointed, with five moderately convex whorls, the first of which is covered with tiny granules, while the rest ones with numerous axial riblets. In its second whorl there is only one spiral keel and in the following whorls two keels narrower than their interspace. The more or less slender teleoconch consists of 11 almost flat whorls. The sculpture consists of spiral cords wider than the interspaces and formed by smooth and rounded pearl-like tubercules which are aligned axially. In the first round of the teleoconch there are only two cords with a third appearing in between them from the sixth whorl and then increasing in thickness. The last whorl narrows sharply at its base with an initially slightly wrinkled cord and subsequently beaded cord in the prolongation of the suture and two more cords between that and the siphonal canal. The aperture bears a simple, somewhat expanded external lip with fragile edge and a deep notch by the suture. The cords by the end of the spire do not divide. The siphonal canal is short, almost closed and forming a conical projection. Very deep chestnut-brown background color all over the shell including the protoconch and its adapical part and much lighter to pale yellowish pearls. The animal had white and translucent tentacules.

**Similar species** *Similiphora triclotae* has a smaller and more slender shell than *Marshallora adversa* (Montagu, 1803), is very similar to *S. similior* (Bouchet & Guillemot, 1978) from which it differs in the white tentacules of the animal in contrast to the black foot and the black line on the tentacles of *S. similior* and from both *M. adversa* and *S. similior* in its pale-yellowish pearls against a dark chestnut-brown background [22].

**Habitat and distribution** In infralittoral rocky bottoms on sponges. Only known from the Strait of Gibraltar and Southern Portugal [22, 27].

**Status** Rare [22].

*Strobiligera flammulata* Bouchet & Waren, 1993 (Figure 4d).

**Collection station** One live individual (8.00 mm in length, 1.55 mm wide) and a juvenile shell (3.05 mm in length, 1.00 mm wide) were collected from detritus material trapped in small scale fishing nets at 120 m depth from mixed bottom of station 14 and at 30 m from mixed bottom of station 15, respectively. One more (first) record by the collector Mr. G. Mpazios from S Greece is discussed in the relevant section.

**Description** The very slender sinistral shell has a high and regularly conical spire. The pointed protoconch bears four very convex whorls, the first of which is covered with tiny intersecting lines while the last two whorls bear a double keel the cords of which are narrower than the interspace and are crossed by numerous, irregular and fine axial riblets. The teleoconch bears 16 almost flat whorls separated by a shallow suture which is difficult to distinguish from the spaces between the cords. The sculpture costists of spiral cords the interspaces of which are much wider than their pointed tubercules which are aligned but not attached axially. At the onset of the teleoconch there are only two cords with a third cord appearing bellow the suture from the fourth whorl and then increasing in thickness. The last whorl bears an additional wrinkled cord in prolongation of the suture. Aperture with a simple lip, a straight columella and a short siphonal canal. Color of randomly arranged alternating cream-white and chestnut-brown areas covering sections of the shell but without conforming to its sculpture.

**Similar species** The species is easily differentiated from the other European Triforids as its first cord of the teleoconch appears on the top of the other two instead of in-between those [22, 27].

**Habitat and distribution** In circalittoral rocky bottoms. Atlantic and W and SW Mediterranean Sea [22, 27].

**Status** Rare [22, 27].

#### Cerithiopsidae

*Cerithiopsis atalaya* Watson, 1885 (Figures 4e and f).

**Collection station** Two shells (6.90 mm and 6.45 mm in length, 1.35 mm and 1.25 mm wide, respectively) were collected from detritus material trapped in small scale fishing nets at 70 m depth from mixed bottom of station 4, and three shells (5.90, 4.95 and 4.50 mm long, 1.15, 0.95 and 0.85 mm wide, respectively) also from detritus material trapped in small scale fishing nets at 25 m from mixed bottom at station 15.

**Description** The shell has a high and regularly conical slender spire. The pointed protoconch bears four highly convex whorls with sculpture formed by two prominent and narrow keels positioned in the middle of the whorls and axial ribs crossed by even thinner, slightly prosocline, ones that extend from suture to suture. The teleoconch bears 14 almost straight whorls, with axial ribs and spiral cords forming cross-shaped pearls in their intersection with the ribs. The first three whorls show two cords with a third one appearing gradually under the suture and progressively increasing in thickness to become equivalent in width to the two others by the last whorl. The pearls of the cords are acute and give the sculpture a rough appearance. The last whorl bears at its base a fourth, more or less, smooth cord below the suture and forms a somewhat concave smooth base. The square aperture has a straight columella, a short siphonal canal and a simple outer lip. The shell exhibits an uneven chestnut-brown color, lighter to yellowish in the first whorls of the teleoconch and in some parts of the later whorls.

**Similar species** *Cerithiopsis atalaya* is very similar to *C. horrida* Monterosato, 1874 from which it differs in that the late is generally larger, its protoconch exhibits a less dense sculpture, the fourth cord of the last whorl is beaded and the pearls are pointed upwards creating a more rough than *C. atalaya* appearance [22].

**Habitat and distribution** In deep rocky bottoms between 80 m and 200 m. Atlantic and SW Mediterranean [22].

**Status** Uncommon [27].

*Cerithiopsis diadema* Monterosato, 1874 (Figure 5a).

**Collection station** One shell (3.50 mm in length, 0.90 mm wide) was collected from biogenic material trapped in small scale fishing nets at 60 m depth from mixed bottom of station 14.

**Description** Shell with high and regularly conical spire. The pointed protoconch is formed by four moderately convex whorls, the first of which bears only very fine spiral striae that extent to the rest three whorls. In addition to that sculptural decoration, these late three whorls are decorated with numerous, somewhat curved, fine axial ribs that extend from suture to suture and are interrupted by a carina which bears a thin spiral cord up to the end of the protoconch. The teleoconch has 10 slightly convex whorls and a fairly deep suture. The sculpture is made up initially of two spiral cords, but from the fourth whorl of the teleoconch a third cord appears just below the suture and increases in width to become equally wide with the other two cords by the last whorl. The cords are approximately of equal width as the interspaces, with smooth and rather flat tubercules that are aligned axially to form ribs. The last whorl diminishes sharply in width by its base and exhibits an additional rather smooth cord in prolongation of the suture. In the proximity of that cord there is a smoother one preceeding a concave area before a short siphonal canal. Light honey color all over the shell including the protoconch.

**Similar species** The very characteristic protoconch in combination with the rest of the shells characteristics separate the species from the superficially similar congeneric and sympatric *C. atalaya* Watson, 1885, *C. fayalensis* Watson, 1885 and *C. horrida* Monterosato, 1874 [22, 29].

**Habitat and distribution** In circalittoral rocky funds of the Atlantic and the W and Central Mediterranean Sea [22, 27].

**Status** Uncommon according to Repetto *et al.*[27] and common based on Gofas *et al.*[28].

*Cerithiopsis fayalensis* Watson, 1886 (Figures 5b and c).

**Collection station** One live individual (3.50 mm in length, 0.90 mm wide) and one shell (3.30 mm in length, 0.85 mm wide) were collected from detritus material trapped in small scale fishing nets at 60 m and 120 m depth, respectively, from mixed bottom of station 14 and two shells (4.15 mm and 3.40 mm in length, 1.35 mm and 1.10 mm wide, respectively) were also collected from detritus material trapped in small scale fishing nets at 20 m depth from mixed bottom of station 1.

**Description** Shell with high and regularly conical spire. The pointed protoconch is formed by five moderately convex whorls decorated with numerous, somewhat curved, fine axial riblets that extend from suture to suture; these riblets are overlaid by an extremely thin and hard to see spiral sculpture. The teleoconch has 8 almost flat whorls with a fairly deep suture. Sculpture made up of three spiral cords, slightly wider than the interspaces, with smooth and rounded tubercules that are aligned axially to form ribs. In the first whorl of the teleoconch, the adapical cord (lower one) is much weaker than the two cords above, then increases in thickness but only in the last whorl it becomes as thick as those two. The last whorl exhibits an additional smooth cord in prolongation of the suture and a smooth concave area between that cord and the short siphonal canal. Light brown to medium-brown color all over the shell including its protoconch.

**Similar species** The very characteristic protoconch in combination with the rest of the shells characteristics separate the species from the superficially similar congeneric and sympatric *C. atalaya, C. horrida* and *C. scalaris*[22].

**Habitat and distribution** In circalittoral rocky funds, exceptionally at 30 m, but principally at 100 m to 300 m, in Atlantic and the W Mediterranean Sea as well as the Ionian Sea [22, 27].

**Status** Uncommon [22].

*Cerithiopsis micalii* (Cecalupo & Villari, 1997) (Figures 5d and e).

**Collection station** One live individual (1.75 mm in length, 0.70 mm wide) and a shell (1.45 mm in length, 0.60 mm wide) were collected from detritus material trapped in small scale fishing nets at 70 m depth from mixed bottom of station 4.

**Description** Shell with cirtoconoid “obese” spire. Protoconch ivory-white, styliform, with four moderately convex whorls bearing flexural axial ribs. Teleoconch with five whorls and a deep suture. Sculpture consisting of tree spiral cords, only two in the first whorl of the teleoconch with a third one formed below the suture and increasing in width in the following whorls. The last whorl which narrows towards its base bears a wrinkled cord as a continuation of the suture and an additional very prominent and also wrinkled one before the siphonal canal. The outer lip of the aperture is simple, fragile and white in contrast to the rest of the teleoconch which is of bright red-brown color.

**Similar species** *Cerithiopsis micalii* has a very similar sculpture and configuration of its last whorl with that of *C. tubercularis* (Montagu, 1803) but is different from the late in the shape of its teleoconch and its characteristic protoconch [22].

**Habitat and distribution** In infralittoral rocky bottoms, presumably on sponges. Atlantic and Mediterranean Sea but only known from a few locations of its western basin [22, 27].

**Status** Rare [22] and uncommon [27].

*Cerithiopsis nana* Jeffreys, 1867 (Figure 6a).

**Collection station** One live individual (4.05 mm in length, 1.25 mm wide) was collected from detritus material trawled at 100 m depth from mixed bottom of station 4, and one shell (3.35 mm in length, 1.05 mm wide) was collected from detritus material trapped in small scale fishing nets at 50 m depth from mixed bottom of station 15.

**Description** Shell with a cirtoconoid spire. The protoconch consists of four slightly convex whorls that form a blunt apex. Just below its suture there is a series of tiny elongated nodules while just over it there is an additional series of nodules forming a very thin cord. The three adapical whorls form a weak carina at their lower part. The teleoconch consists of seven slightly convex whorls. The sculpture is made up of three spiral cords and two more in the last whorl bellow the suture. The upper cord on the spire is weaker than the medium cord and stuck to it, gradually increasing in width and distancing away until by the last whorl it becomes the wider cord. The last whorl narrows at its base, with an extra grainy cord in prolongation of the suture and another one between that and the siphonal canal. The aperture bears a simple, fragile and white outer lip. Light brown color on the protoconch, cinnamon-brown on the spire with brighter the pearls and the additional cords of the base.

**Similar species** *Cerithiopsis nana* is quite similar to *Cerithiopsis tubercularis* (Montagu, 1803) from which it differs in its smaller size, its blunter protoconch and the additional fine cord of tiny nodules over the suture, not present in *C. tubercularis*[22].

**Habitat and distribution** In infralittoral rocky bottoms on sponges. Atlantic and Mediterranean Sea [22].

**Status** Uncommon [27].

*Cerithiopsis* cf. *oculisfictis* Prkic & Mariottini, 2010 (Figure 6b).

**Collection station** Two live individuals (4.05 mm and 4.00 mm in length, 1.15 mm and 1.10 mm wide, respectively) were collected from detritus material trawled at 100 m depth from mixed bottom of station 4.

**Description** The small shell has L/D (length/max diameter) ratio of 3.50, is conical, glossy and slightly scalaroid. Its also conical protoconch (460 μm high, 255 μm wide) is smooth, chocolate-brown in color, semi-transparent, with nearly 4.5 convex whorls and a suture bearing a series of tiny nodules forming a very thin cord. The teleoconch consists of eight, nearly flat, whorls. Its spiral sculpture is composed of three cords made of series of smooth pearls. The adapical cord is initially weaker than the two lower ones but eventually grows equally strong in the last four whorls to become the dominant cord that gives the shell its scalaroid outlook and is distant from the median cord. The body whorl narrows smoothly at its base, with an extra wavy cord in prolongation of the suture and another one between that and the siphonal canal. This last whorl is decorated with 21 ribs weaker than the spiral cords and giving rise to equidistant conspicuous nodules. These nodules become axially ovate on the last three whorls. The suture is deep leading the whorls to be well separated from each other. Aperture sub-quadrangular, smooth and wide. Outer lip simple, thin, orthocline and white. Columellar callus weak. Anal canal broad and short, siphonal canal open and short. Viewed through the aperture, transparency makes visible the sculptural pattern of the spire. Chocolate-brown color on the protoconch, the background of the teleoconch and the base with brighter pearls on the spire. The animal, that quickly withdrew itself into the shell, had a white foot and a dark grey head.

**Similar species** *Cerithiopsis* cf. *oculisfictis* is quite similar to *Nanopsis nana* from which it differs in that the protoconch of *C.* cf. *oculisfictis* bears more convex whorls that lack the weak carina of *N. nana*, the upper cord on the spire is strong in contrast to the weak of *N. nana* and the color is chocolate-brown in contrast to the light brown of *N. nana*[22]. *Cerithiopsis* cf. *oculisfictis* is also similar *C. tubercularis* (Montagu, 1803) from which it differs in that the late has a more slender shell; its protoconch whorls bear a very fine spiral thread just above the suture as well as short axial riblets; the columellar callus is well marked and conspicuously elevated and the propodium is gray to black [32].

**Habitat and distribution** From the intertidal to the upper sublittoral zones (0–8 m), associated to small sponges and known only from certain localities along the Dalmatian coast, North Adriatic Sea [32].

**Status** *Cerithiopsis oculisfictis* is a recently described species and is rather common in the N Adriatic Sea [32].

*Dizoniopsis concatenata* (Conti, 1864) (Figures 6c,d, e and f).

**Collection station** Three live individuals (4.35 mm to 3.00 mm in length, 1.45 mm to 1.00 mm wide) and 3 shells (4.00 mm to 2.50 mm in length, 1.35 mm to 0.90 mm wide) were collected from detritus material trapped in small scale fishing nets at 25 m depth from mixed bottom of station 1. Two shells (3.45 and 2.10 mm in length, 1.15 and 0.70 mm wide, respectively) were collected from detritus material trapped in small scale fishing nets at 30 m depth from mixed bottom of station 15.

**Description** The conical, slightly cyrtoconoid shell has a protoconch with something more than two whorls the first of which is rough with or without minor dotty axial ribs, is globose, narrowing sharply to continue with the second whorl which, in turn, bears on its middle part two close to each other spiral keels. The teleoconch consists of eight almost flat whorls. The sculpture is formed by two cords of about the same width as the interspaces, with rounded pearl-like tubercules. The cord bellow the suture shows pearls with a tendency to widen axially forming elongated tubercules that finally divide as they approach the outer edge of the aperture (*concatenata*). The last whorl narrows at its base and bears two extra cords, a grainy one in prolongation of the suture and a smooth narrower one between the grainy one and the siphonal canal. The outer lip of the aperture is simple, fragile, flairy and white in contrast to the vivid chestnut-red color of the rest of the shell, which becomes paler towards the shells apex.

**Similar species** *Dizoniopsis concatenata* is quite similar to *D. coppolae* (Aradas, 1870) from which it differs in its characteristic protoconch, the dividing pearls of its upper cord by the aperture and by having two additional cords on its last whorl (instead of three in *D. coppolae*). *Dizoniopsis clarkii* (Forbes & Hanley, 1848) and *D. bilineata* (Hornes, 1848) are erroneous identifications [22].

**Habitat and distribution** In infralittoral rocky floors, of unknown with precision hosts and quite often collected live [22]. Known from the Atlantic and the E Mediterranean Sea [22, 27].

**Status** Uncommon [22].

## Discussion

Among the 37 identified species, 16 are referred for the first time for the Hellenic fauna raising its gastropod biodiversity from 631 species [19] and additions by Manousis *et al.*[17] to 651. One more new alien new species of the genus *Emarginula* originated from the Indian Ocean is recorded in the Mediterranean Sea thus enriching significanty (20%) the members of the family Fissurellidae in Greece. Five genera of different families are new for the Hellenic fauna (see Table 1).

The current enrichment of the studied families in the NW Aegean with 16 new species for Greece (with the vast majority of them being minute in size and collected from hard biogenic substrates), 14 of which are new for the E Mediterranean Sea and one of them being new for the Mediterranean Sea, was expected to take place. It is attributed to i) the few and, in some cases, old studies on the gastropod fauna of the area, ii) the lack of search in various environments as far as the different depths and the types of substrate is concerned iii) the collection tactics related to the type of habitats, to all possible and available material and substrate sources (e.g. discarded material from the fishing boats), to the collection equipment, to the detailed sorting of minute in size organisms and to the repeated search efforts and iv) study areas with variable substrates and clean marine environment promise a rich biodiversity.

*Anatoma aspera* (Philippi, 1844) - one of the small and fragile members of the genus *Anatoma -* has been reported by Geiger [26] for the first time from the S Aegean Sea and Sporades in coordinates of ~36° N, 27° E (most probably he means South Sporades - the old name of the Dodecanese - because the coordinates of the name that he uses “Sporades” are ~39° N, 24° E and is located in the N Aegean Sea). The finding of the newly described minute species of *Anatoma micalli* reveals, apart from the research gaps, the identification difficulties on the *Anatoma* species, attributed to numerous misidentifications, the various chresonyms and synonyms mainly of the species *A. aspera*, *A. micalii* and *A. umbilicata*[26] and the lack of useful tools for identification, such as publications with full descriptions and comparisons of the species, and the shortage of detailed and high quality images.

After the collection of *Obesula marisnostri* specimen during this study, three more live individuals and a shell (7.75 to 5.05 mm in length, 2.30 to 1.50 mm wide) (Figure 7a) were collected by the collector Constantinos Kontadakis from biogenic material trapped in small scale fishing nets at 60–100 m depth from mixed bottom of Central Saronikos Gulf (S Greece). The specimen of *Strobiligera flammulata* of this study was collected one month later (26 June 2013) after (26 May 2013) the collector George Mpazios collected one shell (8.90 mm in length, 1.95 mm wide) (Figure 7b) from biogenic material trapped in small scale fishing nets at 80–120 m depth from mixed bottom of SW Saronikos Gulf by Epidaurus (37° 38.000’ N, 23° 11.500’ E). The almost simultaneous findings of *Obesula marisnostri* and *Strobiligera flammulata* both from N and S Greece indicates that i) they are wider distributed in the Hellenic Seas and the Eastern Mediterranean Sea, ii) independent searches from the same type of substrates (e.g. biogenic bottom) and direct sampling might bring to light more species that could otherwise be lost during the fishing procedure and handling and iii) the potential source of information on biodiversity issues through the use of appropriate fora).

The unexpected and simultaneous finding of two live individuals very similar to the species *Cerithiopsis oculisfictis* did not give us the time to examine in detail the color pattern of the living animal and look for the two (characteristic for the species) black spots on the propodium [32], and, therefore, their identification has to remain as *C.* cf. *oculisfictis*. Moreover, at the species level, the color pattern of the head-foot (propodium) comprises a diagnostic feature in the *Cerithiopsis tubercularis* complex [33].

Among the new findings, *Emarginula decorata* - referred from SE Africa [23], the Reunion Islands, Red Sea and the Arabian Gulf [21] - is a new alien mollusc species for the Mediterranean Sea added to the already recorded 215 ones by 2012 [34]. The dispersal of such a benthic organism is attributed to biological (endogenic) and environmental (exogenic) parameters. More specifically, among the biological parameters, the potential migration of a gastropod species in the form of teleplanic larvae could be a result of i) the larvae “escape” degree from the “parent area”, ii) their survival as meroplankton and iii) the chance for reproduction as adults in the new environment [35]. As far as the environment itself is concerned, the parameters include human activities and their effects on the dispersal of the larvae (directly by means of currents and ships and indirectly by means of aquacultures) and the juveniles/adults ratio (directly by means of aquacultures and indirectly by means of transportation as epibenthic/epibionts). Nevertheless, the climate changes in the Mediterranean Sea and the almost 1000 different alien species recorded by 2012 [34] have particularly changed the biodiversity during the last two or three decades having as a result the publication of numerous articles in which the reasons for occurrence of aliens in the Mediterranean Sea, the frequency of the records, the vectors and the distribution pathways have been extensively discussed (e.g. [34, 36–41]).

**Figure 8:**
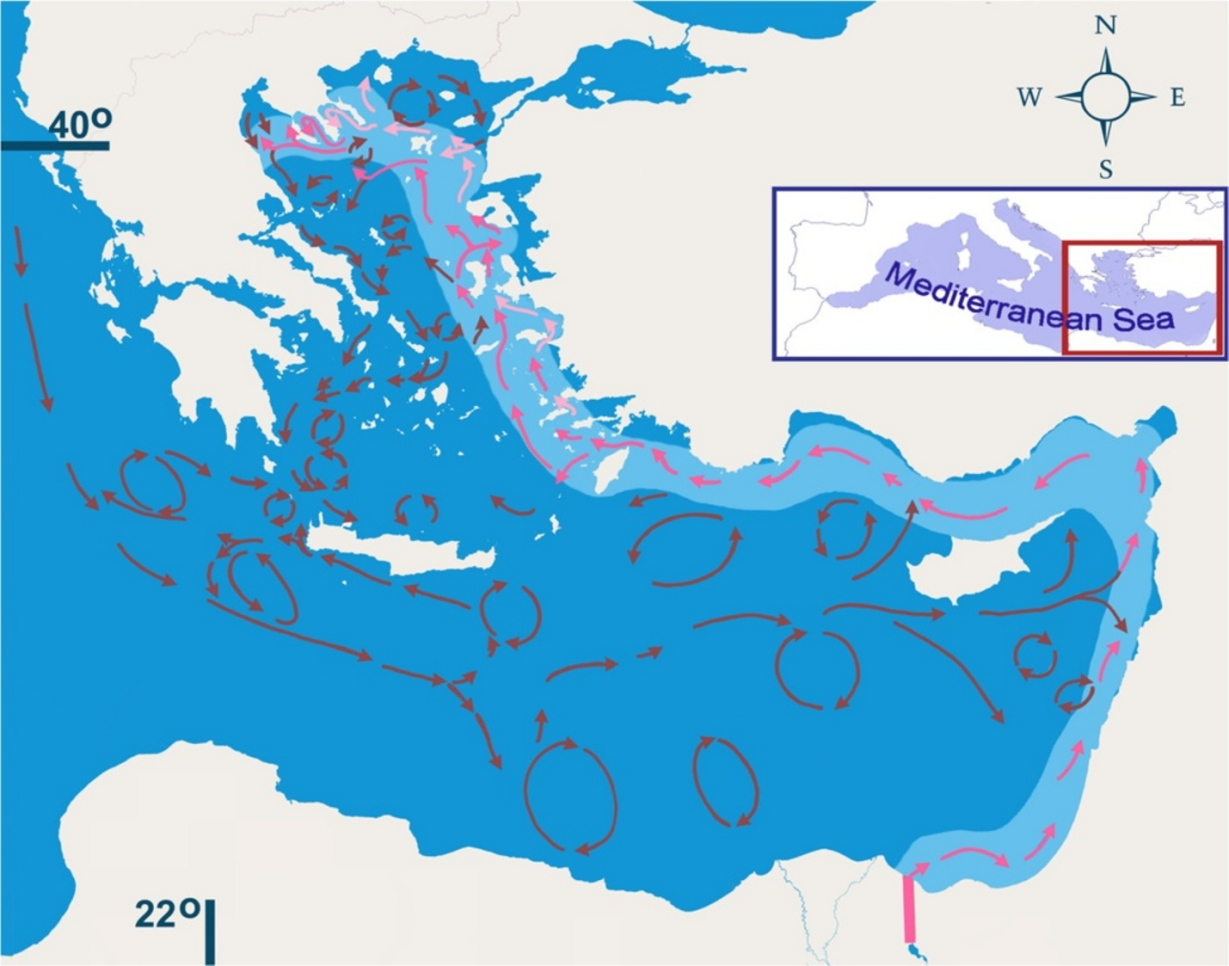
**The sea surface currents in the Eastern Mediterranean Sea.** The pink arrows represent the currents probably responsible for the expansion of the Lessepsian molluscs and the blue thinning zone shows the probable pathway of that expansion from the Suez Canal towards north up to the NW Aegean Sea. Heavy ink arrows indicate the main pathway and the light ones a secondary pathway. Reconstructed map for the currents according to Robinson *et al*. [42], Olson *et al*. [43], Sayin *et al.*[44] and Poulain *et al.*[45].

More than half (54%) of the marine alien species in the Mediterranean have, most probably, entered the area through “corridors” such as - and mainly - the Suez Canal. Shipping is directly connected with the introduction of only 12 species, whereas it is indirectly assumed as being the most probable way for the introduction (via ballasts or fouling) of another 300 species [34]. The alien species *Emarginula decorata* recorded from Siggitikos Gulf indicate that its possible vector is the sea currents rather than the limited navigation in that area. Suez Canal is one of the most significant hot points for alien dispersal to the East and the West [41]. Following the sea currents model in the Mediterranean Sea, the eastern current direction is correlated with the northward progressive dispersal of the Lessepsian molluscs along the coasts of Israel, Lebanon, Syria, and from there towards the southern coasts of Turkey and the coasts of Cyprus, and, finally, in-between the Greek islands of the E Aegean and the Aegean coasts of Turkey [41–45]. Taking, though, into account the directions of the sea currents in the Aegean Sea (Figure 8), the proposed by Tzomos *et al*. [41] corridor along the E Aegean seems to be a rather secondary one for the molluscs of deeper waters, as the main currents move parallel and along the western coasts of the Hellenic islands of the E Aegean Sea. As these currents approach the Dardanelles and due to the Black Sea SW currents of lower salinity waters, they turn west, continue towards the NW Aegean in a pathway of the same higher salinity than that of the NE Aegean Sea, pass south of the Chalkidiki Peninsulas branch and enter Siggitikos and Toronaeos Gulfs and subsequently Thermaikos Gulf (Figure 8).

The work continues on other molluscan families and expands in more areas while collaborations between researchers and collectors could effectively improve the marine biodiversity profiles of the Hellenic Seas.

## Conclusions

Thirty seven species, the majority of which is of minute size, belonging to seven families (Cerithiopsidae, Fissurellidae, Phasianellidae, Scissurellidae, Siliquariidae, Skeneidae, and Triphoridae) were identified. Among those, one (*Emarginula decorata* Deshayes, 1863) is a new alien for the Mediterranean Sea, 14 are new for the Eastern Mediterranean Sea and 16 are new for the Hellenic fauna with the two above mentioned alien species included. The new findings are attributed both to the sampling methods employed and the under- or unsearched marine environments as far as different types of substrates and depths are concerned. Based on the new findings of this study, the pathway of alien species distribution to the N and NW Aegean Sea [46] is extended up to Thermaikos Gulf.

## Methods

The sampling of specimens was conducted from October 2008 to January 2014 in certain locations of Thermaikos, Toronaeos and Siggitikos Gulfs (Figure 1) by i) sieving soft bottom surface of shallow waters through a series of sieves with a mesh of 5 mm, 2 mm and 0.5 mm, ii) diving down to a depth of 10 m of the infralittoral zone, iii) searching the supralittoral of several coasts of the Gulfs and iv) searching only fresh trawled and discarded material from small scale fishing nets taken from the vessels. That particular material from the Toronaeos Gulf was consisting mainly of biogenic substrate pieces, accompanied, in cases, by parts of maerl. After cleaning with fresh water, shells were treated with a small amount of parafine oil in acetone, left for acetone to evaporate and examined under a stereoscope with a magnification of up to 80×. For each species collected, the following data have been recorded: location, depth, type of habitat/substrate and size (length, unless otherwise stated). The species recognition was based on a) systematic guides, atlases such as Delamotte & Vardala-Theodorou [12], Gofas *et al*. [22], Repetto *et al*. [27], Poppe & Goto [46], Giannuzzi-Savelli *et al*. [47], Cachia *et al*. [48–50], Doneddu & Trainito [51], Cossignani & Ardovini [52], b) faunistic and review articles (i.e. [53–55]), c) studies on the Mollusca fauna in the Hellenic seas [17, 56–59].

Information from specific web sites was also taken into account (30 June 2014). More specifically, for the species nomenclature update, besides the Marine Biodiversity and Ecosystem Functioning EU Network of Excellence (MarBEF) [60] and the World Register of Marine Species (WoRMS) [23] the Taxonomic on-line Database on European Marine Mollusca (CLEMAM) [61] was used. In addition, the Ellenic Network on Aquatic Invasive Species (ELNAIS) [62] and the Marine Mediterranean Invasing Alien Species dadabase (MAMIAS) [63] were used for the alien species status in the Hellenic and Mediterranean Seas.

The specimens are deposited in the premises of the Alexander Technological Educational Institute of Thessaloniki and those of Dr. T. Manousis. Scientists are welcome to have access to the biological material at will.
